# Lithotomy and Lithotrity

**Published:** 1872-09

**Authors:** 


*Lithotomy and Lithotrity.* Illustrated by cases in the practice of GORDON BUCK, M. D. New York: Wm. Wood & Co., 1872.

Dr. Buck gives in this monograph an expression of his views in regard to Lithotomy and Lithotrity, strengthened and illustrated by cases which have occurred in his practice during a long series of years.

The author divides his cases into groups, as follows:

Group I. Comprises cases in which the moderate size of the calculus, and a favorable condition of the urethra and bladder, as also of the general system, indicated lithotrity as preferable to lithotomy. Under this head are enumerated twenty-four cases.

Group II. Comprises cases where the stone was large, though soft; the bladder healthy and the urethra capacious—a concurrence of circumstances permitting the successful employment of lithority. Under this head seven cases are cited.

Group III. Comprises cases in which, from the unfavorable condition of the bladder or urethra, or from the large size and hard composition of the calculus, lithotomy should be resorted to in preference to lithotrity.

The cases reported under this group are fourteen in number, and may be arranged as follows:

One case was, after trials by lithotrity, advised to submit to lithotomy, but the operation being postponed till cool weather, the patient died in the mean time. Six cases by lithotrity proved fatal. One case in which ten operations by lithotrity had been performed, was submitted to the operation of lithotomy as a last resort, but this proved fatal. Six cases by lithotomy (two boys) proved successful.

Of the forty-five cases operated upon forty-one were males and four females. Five of the forty-five had relapses, which might fairly be regareded as the result of new formations. In these five cases twelve relapses in all occurred, making an aggregate of fifty-seven cases. "Six, after trial of lithotrity, were abandoned to lithotomy, of whom one declined and five submitted; of these four recovered and one died. Two were relieved and passed out of notice. Thirty-nine recovered. Eight died—seven males, one female."

The observations in regard to the mode of operating, number of operations, etc., etc., are well made and give evidence of thought and observation. That the paper contains much valuable and interesting matter no one who gives it his careful attention can fail of observing.

